# The Conserved MAPK Site in E(spl)-M8, an Effector of Drosophila Notch Signaling, Controls Repressor Activity during Eye Development

**DOI:** 10.1371/journal.pone.0159508

**Published:** 2016-07-18

**Authors:** Mohna Bandyopadhyay, Clifton P. Bishop, Ashok P. Bidwai

**Affiliations:** Department of Biology, West Virginia University, Morgantown, West Virginia, United States of America; National Institutes of Health (NIH), UNITED STATES

## Abstract

The specification of patterned R8 photoreceptors at the onset of eye development depends on timely inhibition of Atonal (Ato) by the Enhancer of split (E(spl) repressors. Repression of Ato by E(spl)-M8 requires the kinase CK2 and is inhibited by the phosphatase PP2A. The region targeted by CK2 harbors additional conserved Ser residues, raising the prospect of regulation via multi-site phosphorylation. Here we investigate one such motif that meets the consensus for modification by MAPK, a well-known effector of Epidermal Growth Factor Receptor (EGFR) signaling. Our studies reveal an important role for the predicted MAPK site of M8 during R8 birth. Ala/Asp mutations reveal that the CK2 and MAPK sites ensure that M8 repression of Ato and the R8 fate occurs in a timely manner and at a specific stage (stage-2/3) of the morphogenetic furrow (MF). M8 repression of Ato is mitigated by halved EGFR dosage, and this effect requires an intact MAPK site. Accordingly, variants with a phosphomimetic Asp at the MAPK site exhibit earlier (inappropriate) activity against Ato even at stage-1 of the MF, where a positive feedback-loop is necessary to raise Ato levels to a threshold sufficient for the R8 fate. Analysis of deletion variants reveals that both kinase sites (CK2 and MAPK) contribute to ‘cis’-inhibition of M8. This key regulation by CK2 and MAPK is bypassed by the *E(spl)D* mutation encoding the truncated protein M8*, which potently inhibits Ato at stage-1 of R8 birth. We also provide evidence that PP2A likely targets the MAPK site. Thus multi-site phosphorylation controls timely onset of M8 repressor activity in the eye, a regulation that appears to be dispensable in the bristle. The high conservation of the CK2 and MAPK sites in the insect E(spl) proteins M7, M5 and Mγ, and their mammalian homologue HES6, suggest that this mode of regulation may enable E(spl)/HES proteins to orchestrate repression by distinct tissue-specific mechanisms, and is likely to have broader applicability than has been previously recognized.

## Introduction

The external sensory organs of Drosophila include the compound eye and the bristles, both of which have served as paradigms to understand mechanisms underlying neurogenesis (reviewed in [1–5]). Studies over the years have revealed many shared principles/processes guiding the development of these sensory organs and those that are distinct. The precise hexagonal (pseudocrystalline) geometry of the Drosophila compound eye [6] has afforded an ideal system to understand the roles of signaling pathways and molecular determinants driving cell fate specification and pattern formation [3, 7–10]. The compound eye of Drosophila is composed of ~750 unit eyes (ommatidia), each of which includes eight photoreceptors (R1-R8), twelve accessory cells and an inter-ommatidial bristle (IOB). During the third larval stage (instar), eye morphogenesis initiates at the posterior margin of the eye anlagen (eye/antennal disc), a neuro-epithelium that is the progenitor of the retina. Retinal neurogenesis starts with the specification of the R8 photoreceptors in a moving wave of differentiation, called the MF [11]. While R8 selection occurs within the MF, all secondary photoreceptor recruitment occurs posterior to it [11, 12]. The temporal nature of the MF thus represents a 48-hour window, encompassing retinal neurogenesis.

R8 specification involves biphasic Notch signaling [13]. At stage-1, the leading edge of the MF, Notch induces low level expression of the proneural bHLH activator Atonal (Ato, [14–16]) to form the ‘Ato-stripe’. Heterodimers of Ato and Daughterless (Da, [17]) then elicit high Ato levels (*ato* auto-activation), from which arise groups of equipotential cells akin to proneural clusters (PNCs, [18]). At stage-2/3 of the MF, the future R8 (from each cluster) expresses high level of Delta (Dl), which activates Notch (N) signaling in adjacent cells, and culminates in Suppressor of Hairless (Su(H)) dependent transcription of the *E(spl)C*-encoded bHLH repressors [13]. The E(spl) proteins along with Gro, restrict Ato expressivity/function to a single cell, the future R8, from each PNC in a process called ‘lateral inhibition’ [13], involving many components that also regulate bristle development [19–22]. By stage-4, patterned ‘founding’ R8 photoreceptors emerge (are born), which are marked by expression of the Ato target *senseless* (*sens*) [23, 24]. The R8s then systematically recruit surrounding uncommitted cells as the R2/R5, R3/R4, R1/R6 photoreceptors, concluding with the specification of the R7 cell [12].

Given that lateral inhibition is key to patterning of the eye and bristles, several studies sought to analyze modes of action of E(spl) proteins [25–29]. These studies have been complicated by the fact that the *E(spl)C*, a genetically dense locus [26, 30–32], encodes seven structurally similar bHLH members that are expressed in overlapping patterns, and because mutations uncovering each individual member have been unavailable. Consequently, deficiencies uncovering multiple members are necessary to elicit ‘neurogenic’ phenotypes [33, 34]. As an alternative, the Gal4-UAS approach [35] has been used, revealing a generalized loss of bristles and their sensory organ precursors (SOPs) upon over-expression of any E(spl) member [28, 29]. These proteins have accordingly been considered to be partially redundant [36, 37], a view extending to their mammalian homologues, the HES proteins. Redundancy, however, seems an oversimplification, as the *E(spl)C* remains largely unchanged over ~100 MYR [38–41], a time scale that includes divergence of Drosophila and the stalk-eyed flies, Teleopsis.

Of relevance to studies reported here, three E(spl) members, M8, Mγ, Mδ, are expressed in the MF during R8 selection, but ectopic expression of only Mδ (at double dose) impairs R8 birth [13]; a single copy elicits no such effects (Jozwick and Bidwai, unpublished). The importance of M8 is nevertheless highlighted by the severe loss of R8s and reduced eye when the unique dominant *m8* allele *E(spl)D* is combined with the gain of function *Notch* allele, *N*^*spl*^ [42]. It was, in fact, this genetic interaction that led to the identification of the *E(spl)C*. The *E(spl)D* mutation encodes M8*, a truncated protein lacking the 56 residue C-terminal domain, the CtD [43, 44]. Importantly, ectopic M8*, but not full length M8, fully mimics the R8/eye defects of *E(spl)D*, indicating that CtD removal elicits hyperactivity. Studies from our laboratory, identifying CtD phosphorylation by protein kinase CK2 revealed key insights into the mechanisms underlying hyperactivity of M8*.

CK2 phosphorylates M8 at Ser159 in the CtD, at a site conserved in M7 and M5 [45]. Replacement of Ser159 with a phosphomimetic Asp residue (M8-S159D) elicits exacerbated loss of R8s and the eye with potency akin to *E(spl)D* [43, 46, 47]. Moreover, M8* and M8-S159D physically interact with Ato with equal binding strength, leading to the proposal that the CtD auto-inhibits full-length M8 [48]. Phosphorylation displaces this intra-molecular (cis) interaction permitting binding to and repression of Ato. The truncation of M8* sidesteps this regulation, implicating CK2 as a regulator of R8 selection [46]. Accordingly, targeted CK2-RNAi elicits the specification of supernumerary R8s and bristle SOPs [49], hallmarks of impaired lateral inhibition.

Follow-up studies revealed that unlike its effects at stage-2/3 of the MF, expression of M8-S159D at stage-1 has no effect on R8 birth or eye development [47]. This result was paradoxical, because Ato levels are lowest at stage-1, and should have been the most sensitive to repression by (non-inhibited) M8. This inactivity raised the prospect that (on its own) CK2 is insufficient to activate M8. If so, are other regulatory enzymes involved? This possibility was suspect as the CK2 site resides in a highly conserved Ser-rich region of M8, termed the phosphorylation domain (P-domain). One of these, the PXSP motif, appears to closely resemble the consensus for modification by MAPK. Like CK2, the MAPK site (PXSP) is conserved in every M8 isoform from Drosophila and Teleopsis [39, 50].

We suspected that the putative/predicted MAPK site may be of importance because a body of work implicates EGFR/MAPK signaling in birth of patterned R8s. First, it has been well documented that cells at stage-2/3 of the MF harbor active (dual-phosphorylated) MAPK [51–54]. This (dpERK) staining is lost in *egfr*^*-/-*^ clones [55, 56] demonstrating that other RTKs (such as Sevenless) are not responsible for activating MAPK. Second, excess (twinned) R8s are specified in clones of *egfr* or its downstream effectors such as *ras* and *raf* [56]. In canonical EGFR signaling, activated MAPK targets the nuclear transcription factors Pointed (Pnt) and Yan [57]. However, no specification of excess R8s is seen in *pnt*^*-/-*^ clones [53]. Finally, active MAPK is cytosolic [52], a location that should prevent its targeting of nuclear-only Pnt or Yan [58]. The target of activated MAPK at stage-2/3 of the MF has remained elusive. We therefore hypothesized that active EGFR/MAPK signaling at stage 2/3 of the MF may target M8 to ensure that repression of Ato occurs at this specific stage of R8 ontogeny.

The studies we describe here sought to evaluate the importance of the predicted MAPK motif in M8 activity. Site-specific and deletion mutants of CK2 and MAPK sites of M8 have been expressed at different stages of the MF to reveal the roles of these two kinase motifs during R8 specification. Our studies indicate that modifications at both CK2+MAPK sites control ‘cis’-inhibition of M8. These studies are consistent with the possibility that EGFR signaling potentiates Notch pathway activity during R8 patterning, that these two signaling pathways do not always act in an antagonistic manner, and suggest a likely molecular target for activated MAPK at the first step in retinal histogenesis.

## Materials and Methods

### Construction of M8 variants

M8 variants with Ala/Asp substitutions at the known phosphorylation site for CK2 (Ser159) and/or that predicted for MAPK (Ser151) were generated by PCR based site-directed mutagenesis. M8 variants harboring deletions of the CK2 (**S**159DCD) or MAPK (P149L**S**P) consensus motifs were generated by inverse-PCR. M8 variants were flanked by EcoRI and BamHI sites 5’ and 3’ to the coding region, respectively. In addition, all M8 variants incorporated a KOZAK consensus (CAAC) immediately 5’ to the ATG codon for efficient expression. All constructs were fully sequenced to confirm the presence of only the intended mutations.

### Germ line transformation

The Ala/Asp variants of M8 targeting the CK2 and/or MAPK sites were cloned into the vector pUAST-attB [59, 60], whereas the deletion variants were cloned into pUAST [61]. Transgenic lines were generated using a commercial embryo injection facility (BestGene, Inc.). All pUAST-attB constructs were integrated using the ϕC31-docking site at 68E on the third chromosome [62]. The transgenic lines were verified by PCR amplification and sequencing of the PUAST-attB construct, and a single molecularly defined line was used in the studies. For the pUAST-constructs, *w+* progenies were identified and the insertion site was mapped by standard approaches using chromosomes harboring dominant markers. In the case of pUAST constructs, 10 independent insertions of M8-ΔCK2 and M8-ΔMAPK were generated, and of these ≥5 have been used in the studies.

### Biochemical analysis

Protein-protein interactions were analyzed by the LexA-based version of the yeast interaction trap [63, 64]. The bait and prey constructs were expressed as C-terminal fusions with DNA-binding domain of LexA and the activation domain (AD) of B42, respectively. The yeast strain EGY048p was used to evaluate protein interactions, as described [45].

### Fly stocks

The Gal4 drivers were obtained from Bloomington Stock Center (denoted by the prefix B, or obtained from the indicated investigators). These drivers are *109*.*68Gal4* (B6479), *h*^*H10*^ *Gal4* (gift from Janice Fischer, University of Texas, ref [65]), and *scaGal4* [29]. The EGFR mutants *egfr*^*f24*^ (B6500), *egfr*^*f2*^ (B2768) and Ellipse aka *egfr*^*Elp*^ or *Elp*^*1*^ (B5144), were obtained from Bloomington Stock Center. *wdb*^*EP3559*^ flies were a gift from Amita Sehgal (University of Pennsylvania).

### Eye and bristle phenotypes

All crosses were performed on standard Yeast-Glucose medium at 24°C, unless indicated otherwise. Fly heads were dehydrated by sequential passage through a graded alcohol series for 24 hours each (25%-50%-75%-100%), and finally passed through Hexamethyldisalinaze. Heads were mounted on EM stubs, dried for 24 hours, sputter coated with gold and examined with a JEOL-6400 scanning electron microscope at an accelerating voltage of 10–20kV. Images were processed with Adobe Photoshop and collated in Adobe Illustrator. The reduced eye was quantified from ≥20–25 adult flies that were photographed using a Leica MZ16 stereomicroscope equipped with a Leica DFC-480 digital camera. Facet numbers were counted, as described [66]. A similar approach was used to determine bristle phenotypes, which were quantified in ≥50 flies of all relevant genotypes. Bar graphs represent mean values for each genotype and error bars represent SD. Eye size (facet counts) and bristle defects were statistically analyzed using student’s *t*-test and ANOVA.

### Immunostaining and confocal microscopy

Eye-antennal imaginal discs were isolated from late third instar larvae and processed as described [67] with minor modifications. After isolation, discs were fixed in 4% paraformaldehyde in 1x phosphate buffered saline (PBS) for 45 minutes at 4°C. After fixation, discs were washed three times with PBS containing 0.3% Triton X-100 (PBS-TX) for 15 minutes each. The discs were incubated for 12–14 hours at 4°C in PBS-TX containing primary antibody. The following antibodies were used; guinea pig anti-Sens (gift from Hugo Bellen, HHMI-Baylor College of Medicine) at a dilution of 1:2000 and mouse anti-ELAV at a dilution of 1:1000 (obtained from the Developmental Studies Hybridoma Bank, created by the NICHD of the NIH and maintained at The University of Iowa). Sens labels differentiated R8s [23], whereas ELAV is a pan-neural marker [68]. Following primary antibody binding, discs were washed three times with PBS containing 0.3% Triton X-100 and then incubated with secondary antibody at room temperature for 2–3 hours. The secondary antibodies (Molecular Probes) used are, goat anti-guinea pig-IgG coupled to Alexa Fluor 633 (1:1000) and goat anti-mouse-IgG coupled to Alexa Fluor 488 (1:1000). The discs were mounted in 60% glycerol, and viewed on an Olympus FluoView (FV100) for confocal imaging. Images were acquired every 1μm along the apico-basal axis of the discs and then compressed as a Z-stack without the removal of any layers. Compressed Z-stacks were exported as TIFF files and collated in Adobe Illustrator. At least 10–15 discs of the indicated genotypes were stained, and images shown are representative discs.

## Results

### M8 repressor activity in the developing eye requires multi-site phosphorylation

**Fig 1:**
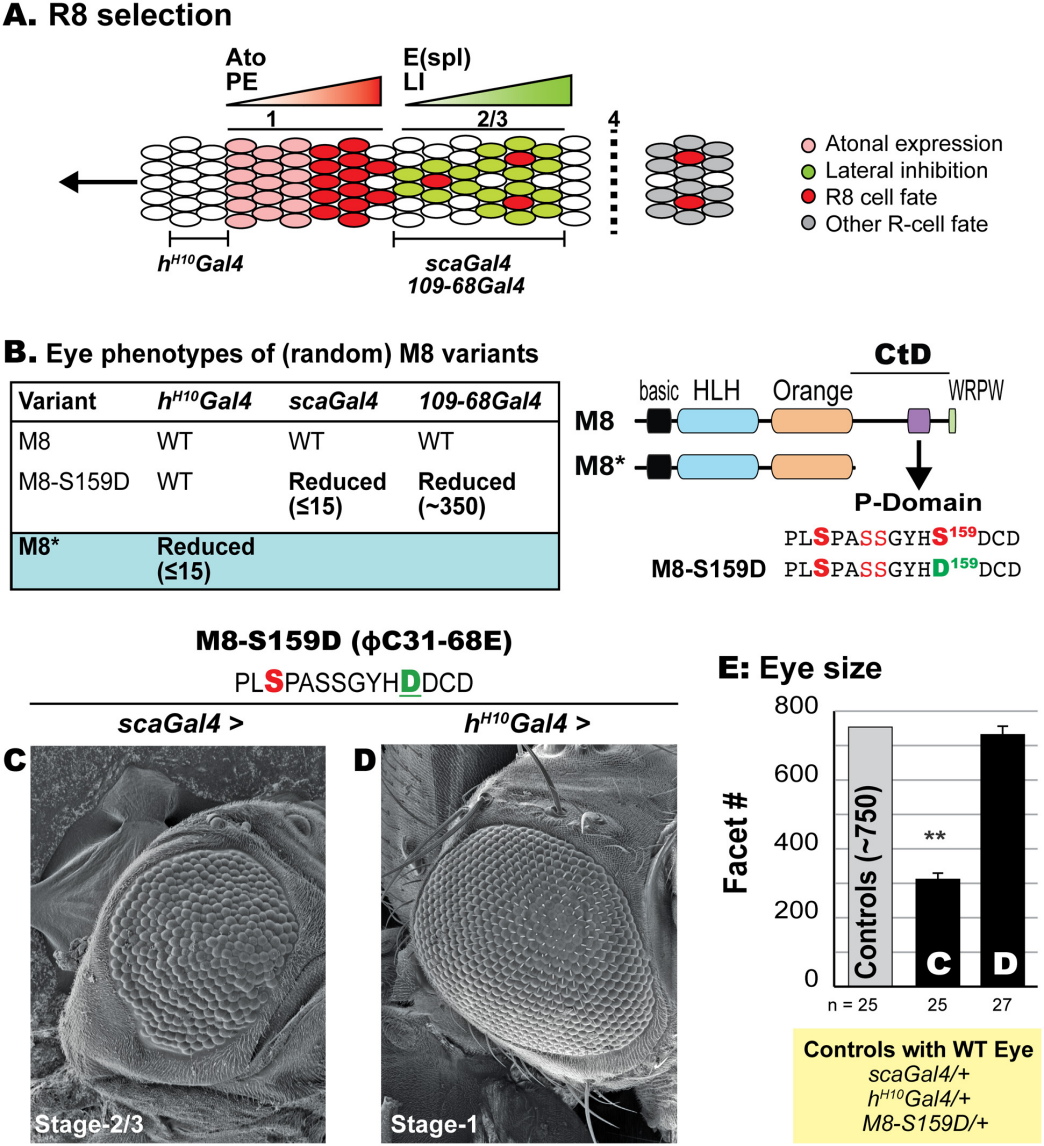
R8 selection and MF specificity of the CK2 mimic M8-S159D. (A) Cell fate acquisition at different stages of the MF. Ato expression is upregulated in response to proneural enhancement (PE), which is followed by lateral inhibition (LI) through the E(spl) repressors. Color codes of cell fates is shown in inset, and expression domains of Gal4 drivers used in the studies are indicated relative to stages of the MF; the vertical dashed line denotes sequential recruitment of secondary photoreceptors. (B) The reduced eye phenotype of CK2 variants (random insertions) or M8*, the product of the *E(Spl)D* allele, upon expression at stage-1 (*h*^*H10*^*Gal4*) and at stage-2/3 (*scaGal4* and *109-68Gal4)*. The reduced eye is denoted by number of ommatidia (facets) remaining; WT denotes facet counts between 750–800 and no perturbation of the hexagonal architecture of the adult eye. Note that the reduced eye of M8-S159D only manifests at stage-2/3, whereas that of M8* occurs at stage-1. Schematic to the right of panel B depicts the domains of E(spl)-M8 and CtD deletion in M8*, and sequence of the P-domain highlighting the CK2 site (S159DCD) that was altered to generate the CK2 mimic M8-S159D. The domains are indicated and include a C-terminal WRPW motif (Gro-binding). (C, D) Scanning EM of the adult eye at 200x. Overexpression of the CK2 mimic M8-S159D elicits a reduced eye at stage-2/3 of the MF (C), but not at stage-1 (D). (E) For each genotype, the number of images analyzed for eye size (ommatidial/facet counts) is indicated. Genotypes shown in panels C and D were compared to controls, and ** denotes P-value < 0.001.

Birth of patterned R8s (Fig 1A) involves three phases of *ato* transcriptional control [15, 18, 69, 70]. The broad and low-level Ato expression initiates at stage-1, is followed by proneural enhancement that raises Ato levels forming clusters of pre-R8 cells, and culminates with its restriction to single R8s by lateral inhibition. The importance of E(spl)-M8 is highlighted by the *E(spl)D* mutation that elicits severe loss of R8s and the eye, phenotypes fully mimicked by stage-1 expression of a UAS-M8* transgene (Fig 1B). Identical effects are elicited by the CK2 phosphomimetic variant M8-S159D (Fig 1B) upon expression at stage-2/3 with either *scaGal4* or *109-68Gal4* (also called *sca*^*109-68*^*Gal4*) whose expressivity correlates to endogenous *E(spl)* genes (Fig 1A). The difference in severity of the reduced eye of M8-S159D (Fig 1B) reflects stronger strength of *scaGal4*, as compared to *109-68Gal4* [71]. As reported [29, 43, 46, 48], wild type M8 elicits no reduced eye (Fig 1B).

Follow-up studies suggest that (on its own) an Asp at the CK2 site does not suffice to activate M8. First, expression of M8-S159D at stage-1 (*h*^*H10*^*Gal4*) is without effect (Fig 1B), an unexpected outcome as Ato levels are lowest at this point of R8 birth (Fig 1A), and should thus have been most sensitive to repression by (non ‘cis’-inhibited) M8-S159D. This inactivity does not reflect weak strength of *h*^*H10*^*Gal4*, as expression of M8* with this Gal4-driver elicits a near complete loss of the eye (Fig 1B). Second, the reduced eye of M8-S159D is strongly mitigated by increased activity of the phosphatase PP2A [50], an unexpected finding as the Asp replacement should have rendered M8 refractory to phosphatase activity. We therefore hypothesized that multi-site phosphorylation of the P-domain (see Fig 1B) controls M8 repression of Ato.

We adopted site-specific (ϕC31) integration to enable controlled comparisons of Ala/Asp variants at multiple kinase sites. We chose the ϕC31 site at 68E, one of ‘moderate’ expressivity [62, 72], as random insertions of M8-S159D elicit a near complete loss of the eye (Fig 1B), likely to be a limit phenotype and thus unsuitable to evaluate multi-site phosphorylation. As expected, the M8-S159D insertion at ϕC31-68E (Fig 1C, 1D and 1E) elicits a moderately reduced eye upon expression at stage-2/3 (*scaGal4*), but not at stage-1 (*h*^*H10*^*Gal4*), effects that qualitatively mimic random insertions.

### E(spl)-M8 harbors a highly conserved consensus site for MAPK

**Fig 2:**
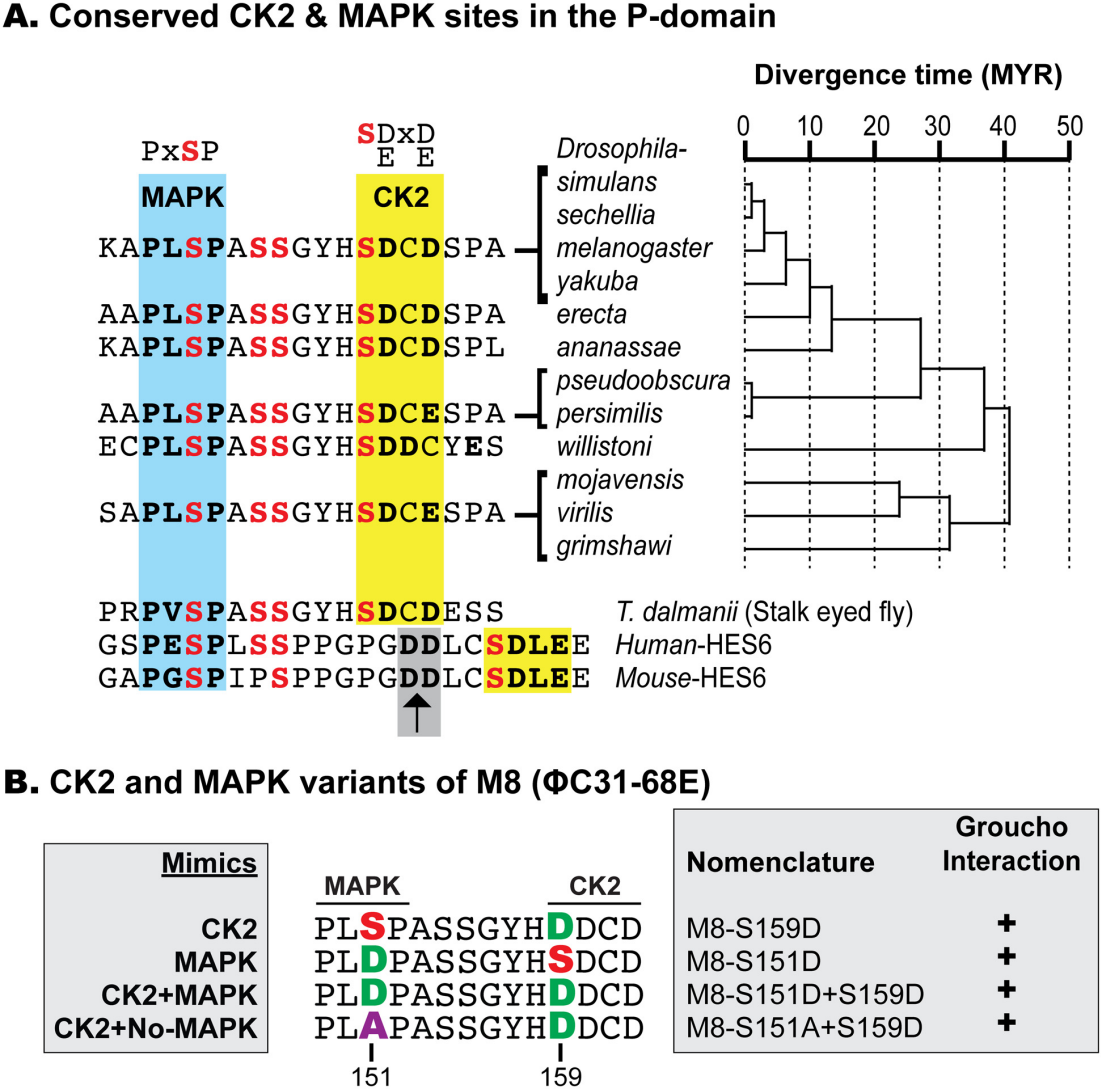
Conservation of Ser residues in the P-domain of E(spl)-M8. (A) Alignment of the P-domain of E(spl)-M8 from the indicated Drosophila species, stalk-eyed flies *Teleopsis Dalmanii*, and mammalian HES6. The evolutionary tree depicts the divergence time of Drosophila species; MYR denotes 10^6^-years. The blue and yellow shaded boxes denote the consensus for MAPK and CK2, respectively (shown at top of alignment). Conserved Ser residues are in bold red. Note that mammalian HES6 harbors six additional residues in the linker separating the MAPK and CK2 sites, and this region harbors the Asp-Asp (DD) motif in the CtD (grey box and black arrow). (B) Transgenic lines for CK2 and MAPK variants were generated using ΦC31 and inserted at site 68E. The nomenclature and predicted behavior of the M8 variants are indicated. Inset on right shows yeast two-hybrid interactions with Groucho; “+” denotes productive interaction.

The P-domain of M8 contains four Ser residues, which are invariant in 12 Drosophila species and the stalk-eyed fly Teleopsis (Fig 2A) that diverged ~100 MYA. Two of these meet the strict consensus for CK2 and MAPK, and the six intervening residues contain two additional phosphoacceptors. In contrast to the CK2 site, which is supported by biochemical studies [45], we consider the PXSP motif to be a potential MAPK site because it meets the consensus for recognition by this family of protein kinases. Both kinase sites are also seen in mammalian HES6, a protein that is modified in its P-domain by CK2 and MAPK [73, 74]. However, the P-domain of HES6 displays three differences with respect to that in the insect M8 proteins. First, an insertion of six additional residues widens the spacing of the CK2 and MAPK sites. Second, this insertion includes tandem Asp residues (DD motif, arrow in Fig 2A), which often bypass the need for phosphorylation. Third, mouse HES6 replaces a Ser with Pro, suggesting that this site may be dispensable in the regulation of human HES6. The full analysis of the contributions of all four Ser residues of M8 is beyond the scope of a single study. We focused on the putative MAPK site, as this effector of EGFR signaling has been implicated in birth of patterned R8s (see Introduction), and because murine HES6 is modified by CK2 and MAPK [73, 75]. The developmental roles of HES6 phosphorylation by CK2 or MAPK remain unknown.

We previously reported that the CK2-refractory variant (M8-S159A) does not mimic unmodified M8, but elicits a rough eye due to dominant-negative (DN) activity [46]. We thus refrained from mutating the MAPK site on a backbone with an Ala at the CK2 site. Variants of the MAPK site (Ser151), were generated using wild type M8 or M8-S159D (Fig 2B). M8-S151A+S159D is refractory to MAPK but mimics CK2, and should reveal if M8 activity requires an intact MAPK site. M8-S151D is a MAPK-mimic that should render M8 independent of EGFR/MAPK signaling, but without modification by CK2. The third, M8-S151D+S159D, is a dual kinase mimic that should render M8 independent of CK2 and MAPK. In a yeast two-hybrid assay (Fig 2B), all variants interact robustly with Groucho.

### An intact MAPK site is essential for activity of the CK2 mimic M8-S159D

**Fig 3:**
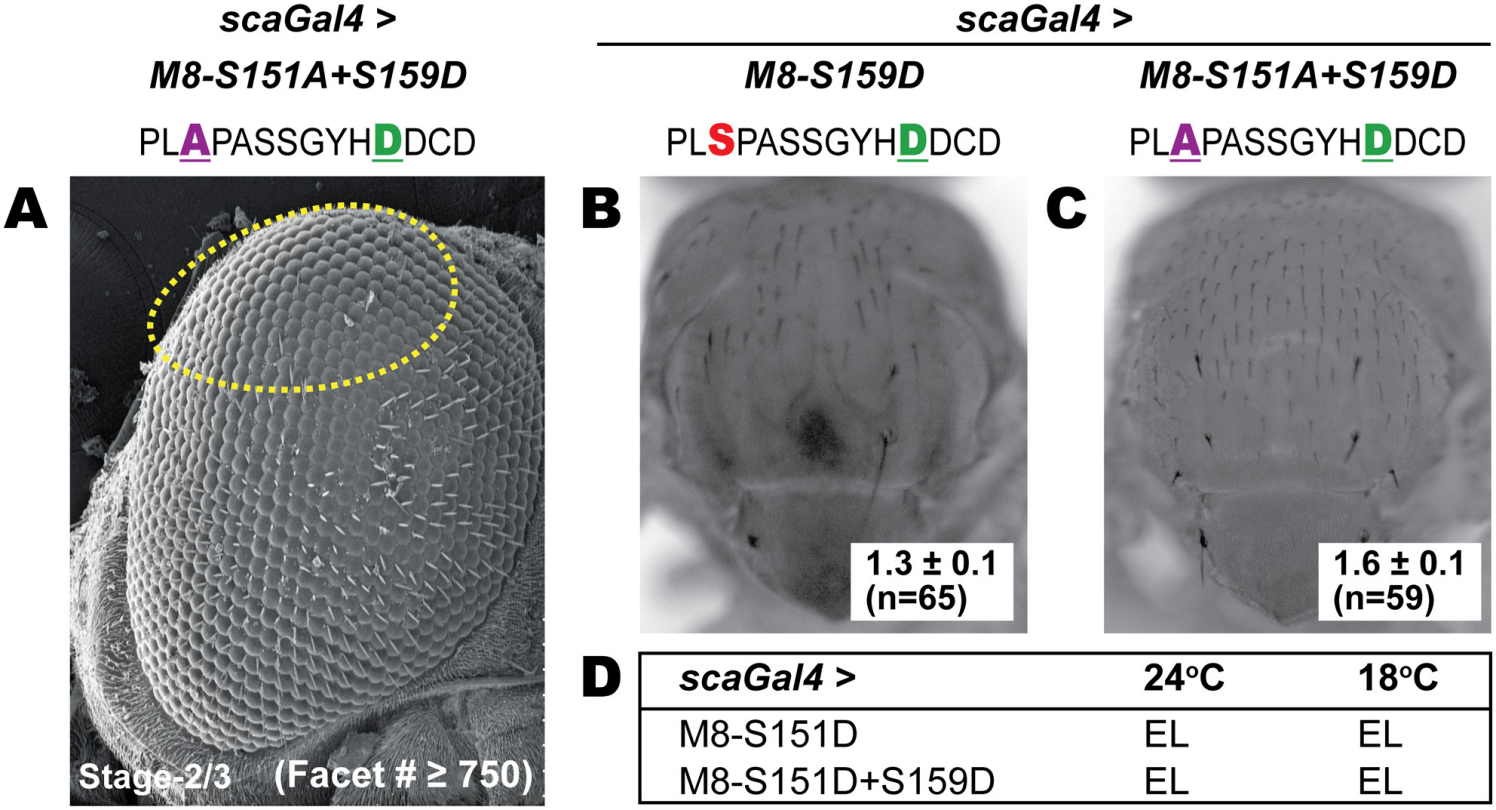
An Ala residue at the MAPK site neutralizes the reduced eye of M8-S159D, but permits bristle suppression. (A) Scanning EM of the adult eye at 200x magnification. The CK2 mimic with an Ala at the MAPK site (M8-S151A+S159D) fails to elicit a reduced eye upon expression at stage-2/3 of the MF. The facet counts (inset) are equivalent to those in wild type flies (not shown). Note that M8-S151A+S159D elicits loss of many IOBs (dashed oval). (B, C) Nota of flies of the indicated genotypes exhibiting loss of MCs. Inset in panels B and C denote MCs remaining per heminotum. (D) The MAPK mimic (M8-S151D) and the dual-kinase mimic (M8-S151D+S159D) elicit embryonic lethality (EL) upon expression with *scaGal4* at either 24°C or 18°C, reflecting expression in most PNCs.

We first compared the activity of M8-S159D (CK2 mimic) and M8-S151A+S159D (refractory to MAPK but mimics CK2). Unlike M8-S159D, expression of M8-S151A+S159D with *scaGal4* did not elicit any reduced eye (compare Figs 3A to 1C). Two findings argue against a defective construct or instability of M8-S151A+S159D protein in vivo. This variant inhibits development of the IOBs (see dashed oval in Fig 3A) and elicits loss of macrochaetes (MCs) and microchaetes (mcs) with potency similar to the CK2 mimic M8-S159D (Fig 3B and 3C). These effects reflect expression of *scaGal4* in PNCs that give rise to these bristle types [28, 29]. Thus an intact MAPK site is essential for M8 activity in the eye, but not the bristle.

We next tested the MAPK-mimic (M8-S151D) and the dual-kinase mimic (M8-S151D+S159D). However, both elicited embryonic lethality when expressed with *scaGal4* at 24C or at 18C where Gal4 activity is attenuated (Fig 3D). Lethality reflects expressivity of *scaGal4* in most PNCs [28].

**Fig 4:**
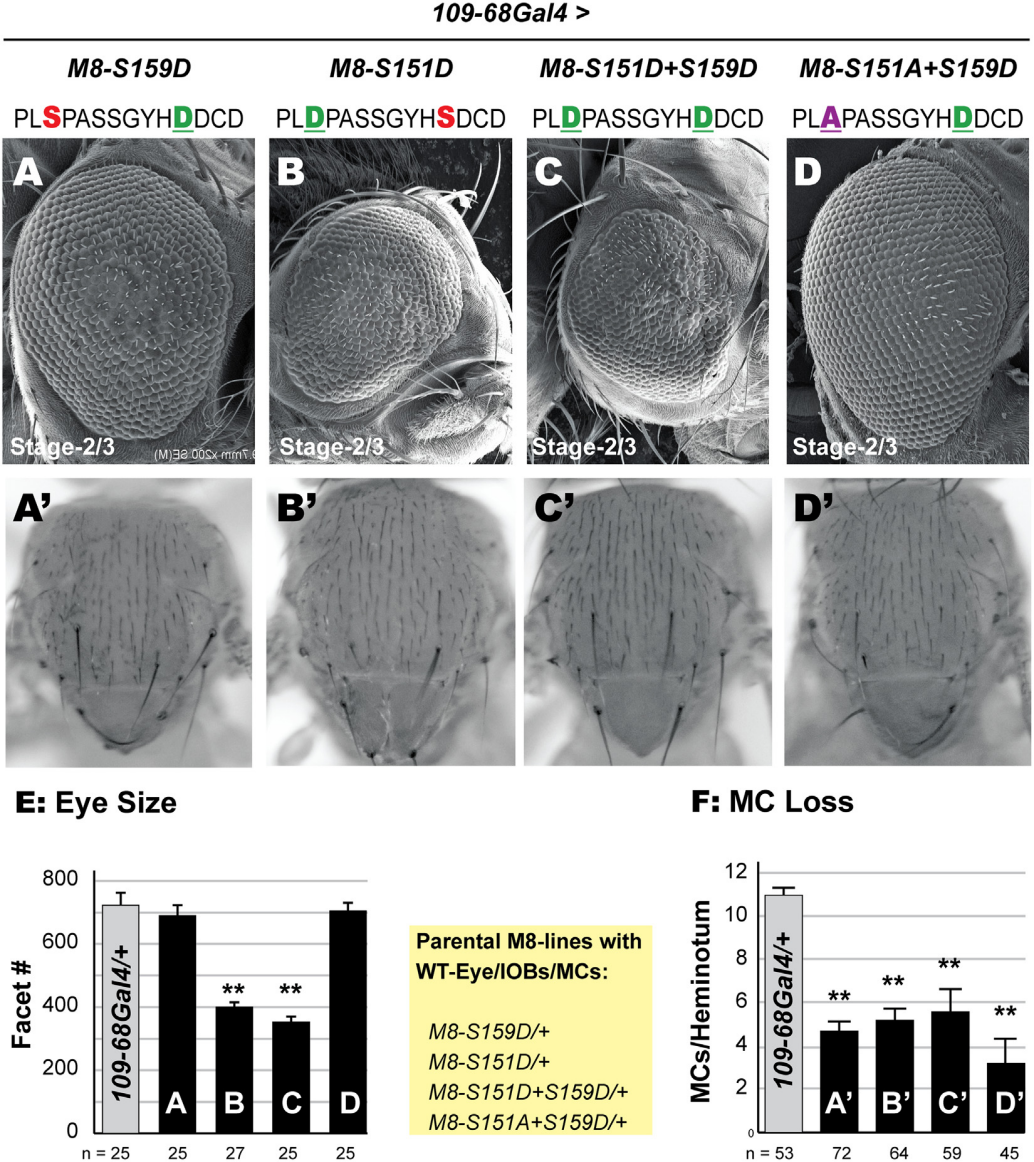
M8 variants with an Asp at the MAPK site elicit a reduced eye. (A-D) Scanning EM of the adult eye at 200x magnification. M8 variants were expressed with *109-68Gal4* at stage-2/3 of the MF. Unlike *scaGal4* (Fig 3), reduced expressivity (strength) of *109-68Gal4* in PNCs prevents embryonic lethality of the MAPK variants, and diminishes the reduced eye of the CK2 mimic M8-S159D (A). In contrast the MAPK-refractory protein M8-S151A+S159D elicits no reduced eye (D), whereas the MAPK-mimic M8-S159D (B) and the dual-kinase mimic M8-S151D+S159D (C) elicit a reduced eye. (E) For each genotype, the number of images analyzed for eye size (ommatidial/facet counts) is indicated. Genotypes shown in panels A-D were compared to control (*109-68Gal4/+*), and ** denotes P-value < 0.001. (A’-D’) Nota of flies expressing M8 variants indicated above panels A-D with *109-68Gal4*. All M8 variants elicit MC loss with almost equal severity (F). For each genotype, MCs (per heminotum) were counted in the indicated number of flies. Genotypes shown in panels A’-D’ were compared to control (*109-68Gal4/+*), and ** denotes P-value < 0.001. Yellow inset indicates that the parental M8-lines had a wild-type eye, IOBs and MCs.

To circumvent lethality, we used *109-68Gal4*, a weaker stage-2/3 driver. Flies expressing all four M8 variants were viable and elicited a range of eye defects (see Fig 4). Expression of the CK2 mimic (M8-S159D) elicited a moderately rough eye and loss of the IOBs, but did not significantly reduce eye size (compare Figs 4A to 1C). This lack of a reduced eye reflects moderate expressivity from the ϕC31-68E site combined with the weaker driver *109-68Gal4*. The rough eye of M8-S159D was abrogated by the simultaneous introduction of an Ala residue at the MAPK site (Fig 4D), although loss of IOBs remained. In contrast, the MAPK-mimic (M8-S151D) or the dual-kinase mimic (M8-S151D+S159D) elicited a reduced eye with equal severity (Fig 4B, 4C and 4E) demonstrating that an Asp at the MAPK site enhances M8 activity in the developing eye. No eye/IOB defects are seen in control, (*109-68Gal4/+*) flies or the parental M8 transgenic lines (inset in Fig 4E). As *109-68Gal4* is active in PNCs that give rise to bristles, we also quantified loss of the MCs, and found that all four variants elicited MC loss (Fig 4A’, 4B’, 4C’ and 4D’) with similar potency (Fig 4F). The lack of effects on the mcs reflects weaker strength of *109-68Gal4*. Together, these studies reinforce the view that the MAPK site is important for M8 activity in the eye, but not the bristle.

### M8 variants with a modified MAPK site perturb R8 specification

We next stained eye discs for Sens and ELAV to evaluate if M8 variants perturb birth of R8s and secondary photoreceptors, respectively. The reduced eye upon expression of the CK2-mimic M8-S159D with *scaGal4* (Fig 1C) reflects strong loss of R8s (Sens+ cells), and many of the R8s that emerge from the MF poorly sustain Sens expression, and inefficiently recruit secondary (ELAV+) photoreceptors [48, 50].

**Fig 5:**
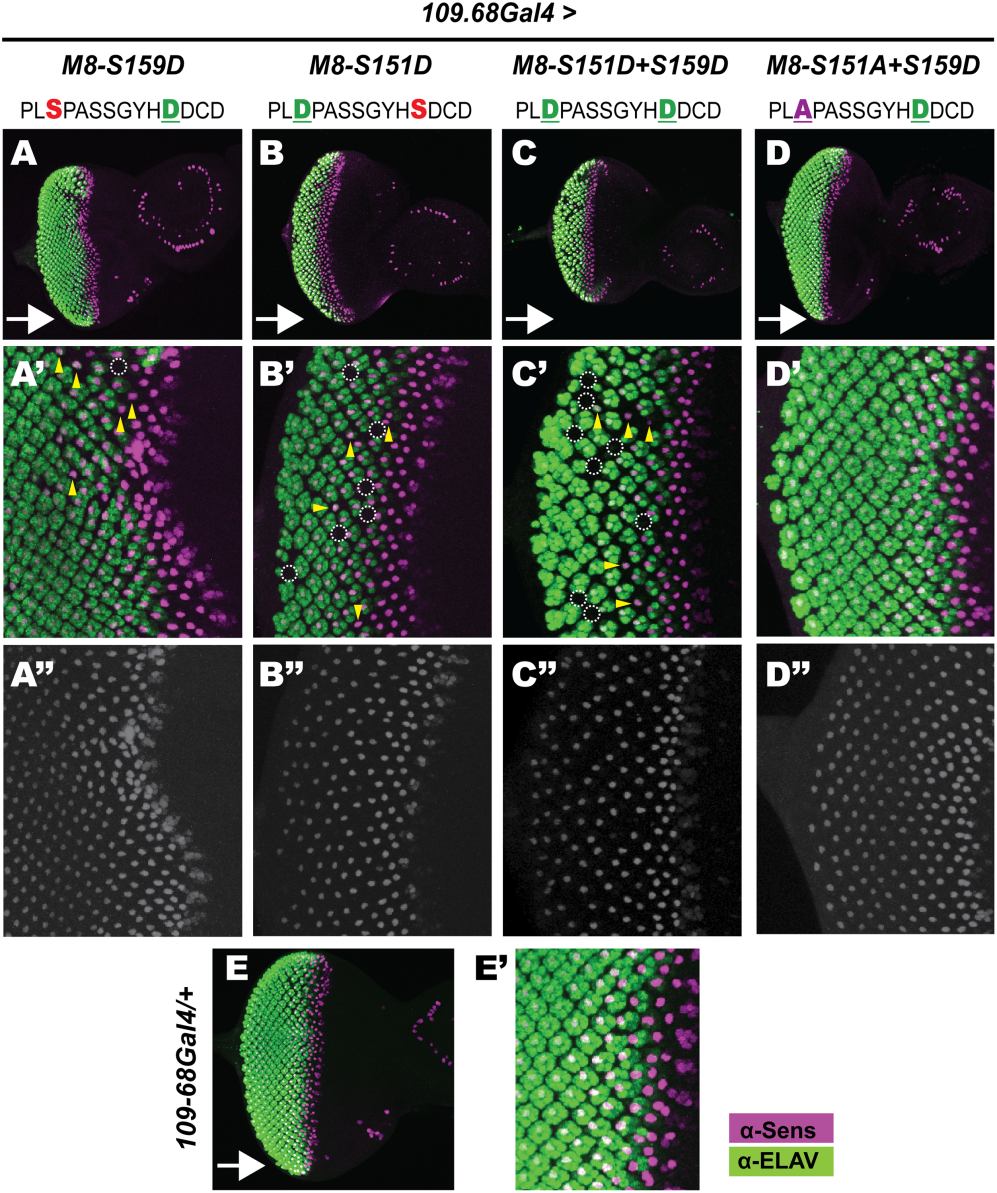
M8 variants with Asp at the CK2 and MAPK sites perturb R8 birth. Eye discs were stained with α-Sens and α-ELAV to label differentiated R8s and secondary photoreceptors, respectively. Arrows indicate direction of MF, dotted circles denote missing R8s and secondary photoreceptors, and arrowheads denote recruitment defective R8s. (A) Discs expressing the CK2-mimic M8-S159D show sporadic gaps in Sens+ELAV clusters, whereas discs expressing the MAPK-refractory variant M8-S151A+S159D (D) closely resemble *109-68Gal4/+* (E) or WT disc (data not shown). Both, the MAPK mimic M8-S151D (B) and dual kinase (CK2+MAPK) mimic M8-S151D+S159D (C) exhibit areas lacking Sens+ELAV clusters with the greatest severity. (A’-D’) magnified view of panels A-D, and (A”-D”) Sens channel in greyscale.

As expression of Asp variants of the MAPK site with *scaGal4* was lethal (Fig 3D), we used *109-68Gal4* to enable side-by-side analysis of all M8 variants. In wild type or *109-68Gal4/+* flies (Fig 5E and data not shown), specification of R8 photoreceptors occurs in a relatively precise manner such that one column of R8s is out of phase with adjacent columns (Fig 5E’), and these R8s maintain Sens expression at relatively constant levels along the AP-axis of the eye disc (Fig 5E and 5E’). In addition, the number of secondary photoreceptors recruited by each R8 to the developing ommatidium progressively increases along the AP axis reflecting progress towards completion of retinal neurogenesis. Thus Sens and ELAV staining reveals R8 patterning and differentiation, and their ability to complete retinal histogenesis. Expression of the CK2 mimic M8-S159D elicited the weakest effects (Fig 5A, 5A’ and 5A”) wherein a few R8s failed to maintain Sens expression (dashed circle in Fig 5A’) and poorly recruited ELAV+ cells (arrowheads in Fig 5A’). Consequently, a few regions of the eye disc are devoid of juxtaposed ELAV clusters (dashed circle in Fig 5A’), as compared to control discs (Fig 5E’). These R8s are likely removed by apoptosis, a default fate for uncommitted cells [76], contributing to the ‘rough eye’ of M8-S159D expressing flies (Fig 4A). These R8 defects are rarely seen in discs expressing M8-S151A+S159D (Fig 5D, 5D’ and 5D”), confirming that an Ala at the predicted MAPK site renders M8 inactive (in the eye) even in the presence of an (activating) Asp at the CK2 site. In contrast, discs expressing the MAPK mimic M8-S151D or the dual-kinase mimic M8-S151D+S159D display inconsistent maintenance of Sens along the AP axis in a greater proportion of R8s (Fig 5B, 5B”, 5C and 5C”). Many of these R8s poorly recruit secondary photoreceptors, such that numerous clusters contain less than the normal 1Sens+7ELAV cells (Fig 5B’ and 5C’). Moreover, regions of the eye disc altogether lack Sens+Elav clusters, a result consistent with the reduced eye. Thus the reduced eyes of the variants described in Fig 4 reflect defective birth and survival of R8s, and impaired secondary photoreceptor recruitment.

### An Asp residue at the MAPK site elicits inappropriate earlier activity of M8

We next tested if an Asp at the MAPK site renders M8 prematurely active, i.e., at stage-1 of the MF. This study was conducted because M8* (lacking the ‘cis’-inhibitory CtD) elicits a strong loss of the eye at stage-1, whereas the CK2 mimic M8-S159D is without effect (Fig 1B and 1D). As (endogenous) MAPK is normally activated at stage-2/3 of the MF where R8s are selected by lateral inhibition, we reasoned that an Asp at the predicted MAPK site might bypass the need for EGFR/MAPK signaling, and enable premature activity at stage-1, akin to M8*.

**Fig 6:**
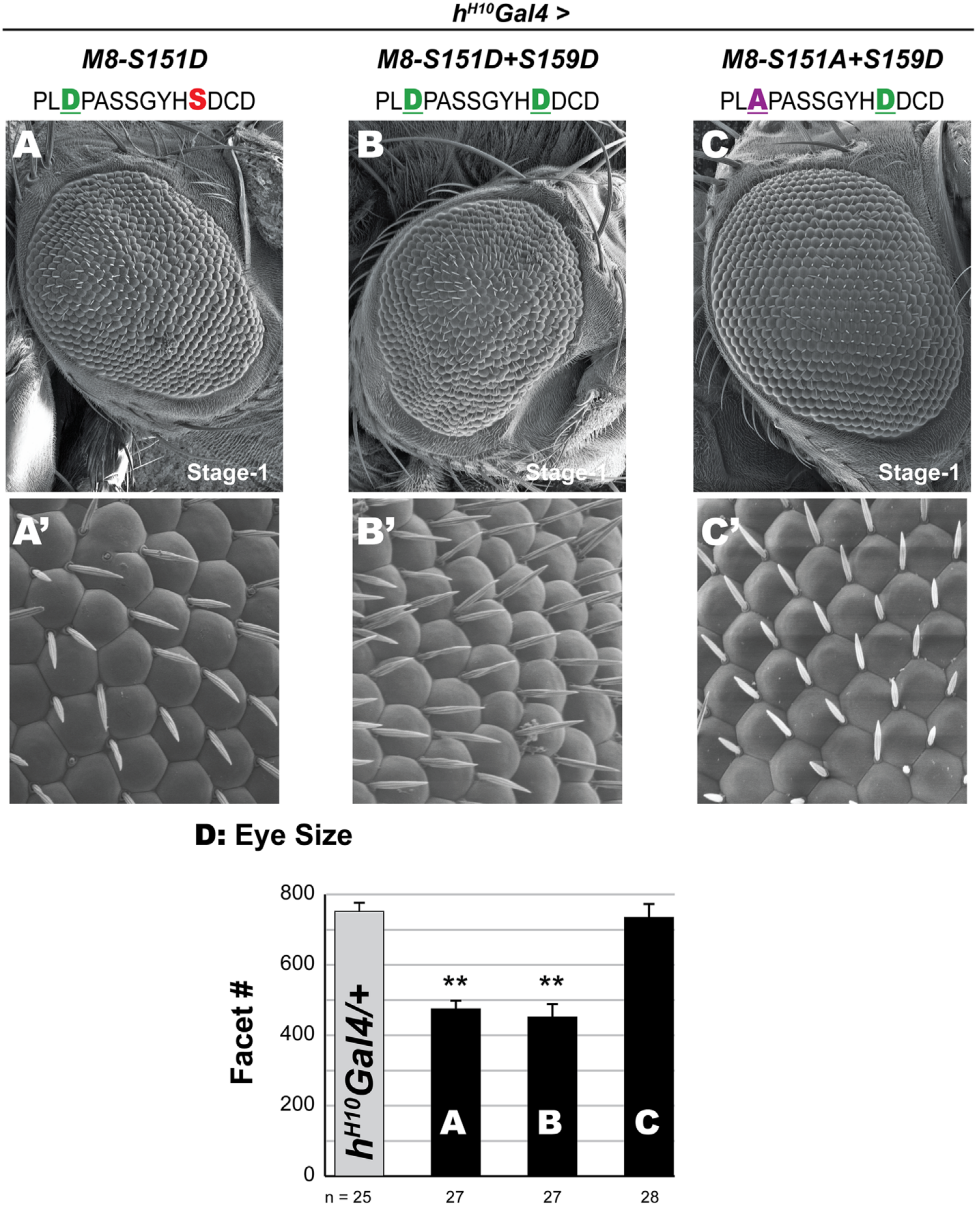
An Asp at the MAPK site engenders M8 activity at stage-1 of the MF. (A-C) Scanning EM of adult eye at 200x magnification. The MAPK mimic M8-S151D (A) and the dual-kinase mimic M8-S151D+S159D (B) elicit a reduced eye, whereas a WT eye is evidenced upon expression of M8-S151A+S159D, a variant refractory to MAPK (C). (A’-C’) 1000x magnification of the eyes in panels A-C. Note that M8 variants with an Asp at the MAPK site elicit the specification of extra IOBs (A’, B’), whereas such defects are infrequently seen upon expression of M8-S151A+S159D (C’). (D) For each genotype, the number of images analyzed for eye size (ommatidial/facet counts) is indicated. Genotypes shown in panels A-C were compared to control (*hH*^*10*^*Gal4/+*), and ** denotes P-value < 0.001.

Indeed, stage-1 expression (*h*^*H10*^*Gal4*) of the MAPK-mimic (M8-S151D) or the dual-kinase mimic (M8-S151D+S159D) elicits a reduced eye (Fig 6A and 6B) with similar potency (Fig 6D). Additionally, both variants perturb the ommatidial lattice and the position of the IOBs (Fig 6A’ and 6B’). No reduced eye was evinced with M8-S151A+S159D (mimics CK2, but not MAPK), and neither did this variant perturb the ommatidial lattice or the position of the IOBs (Fig 6C, 6C’ and 6D). These data seem consistent with the possibility that both CK2 and MAPK are needed for proper activation of M8 in a timely manner at stage-2/3. Importantly, earlier (stage-1) activity is engendered by Asp mutations at the predicted MAPK site, which mimic CtD deletion in M8*.

### Altered EGFR signaling modulates M8 variants with an intact MAPK site

We next tested for a role for EGFR signaling by employing *egfr*^*f24*^, a loss of function allele, which does not perturb the eye/R8s in the heterozygous state (data not shown), but is lethal when homozygous. The expectation was that if EGFR/MAPK signaling were to activate M8, then halved EGFR levels might attenuate the amount of active MAPK, and diminish phosphorylation of M8. If so, M8-S159D (CK2 mimic with an intact MAPK site) should become hypo-phosphorylated in *egfr*^*f24*^*/+* flies, dampening the severity of its reduced eye. In contrast, the MAPK-mimic (M8-S151D) and the dual-kinase mimic (M8-S151D+S159D) should be refractory to halved EGFR levels by virtue of the Asp at the MAPK site.

**Fig 7:**
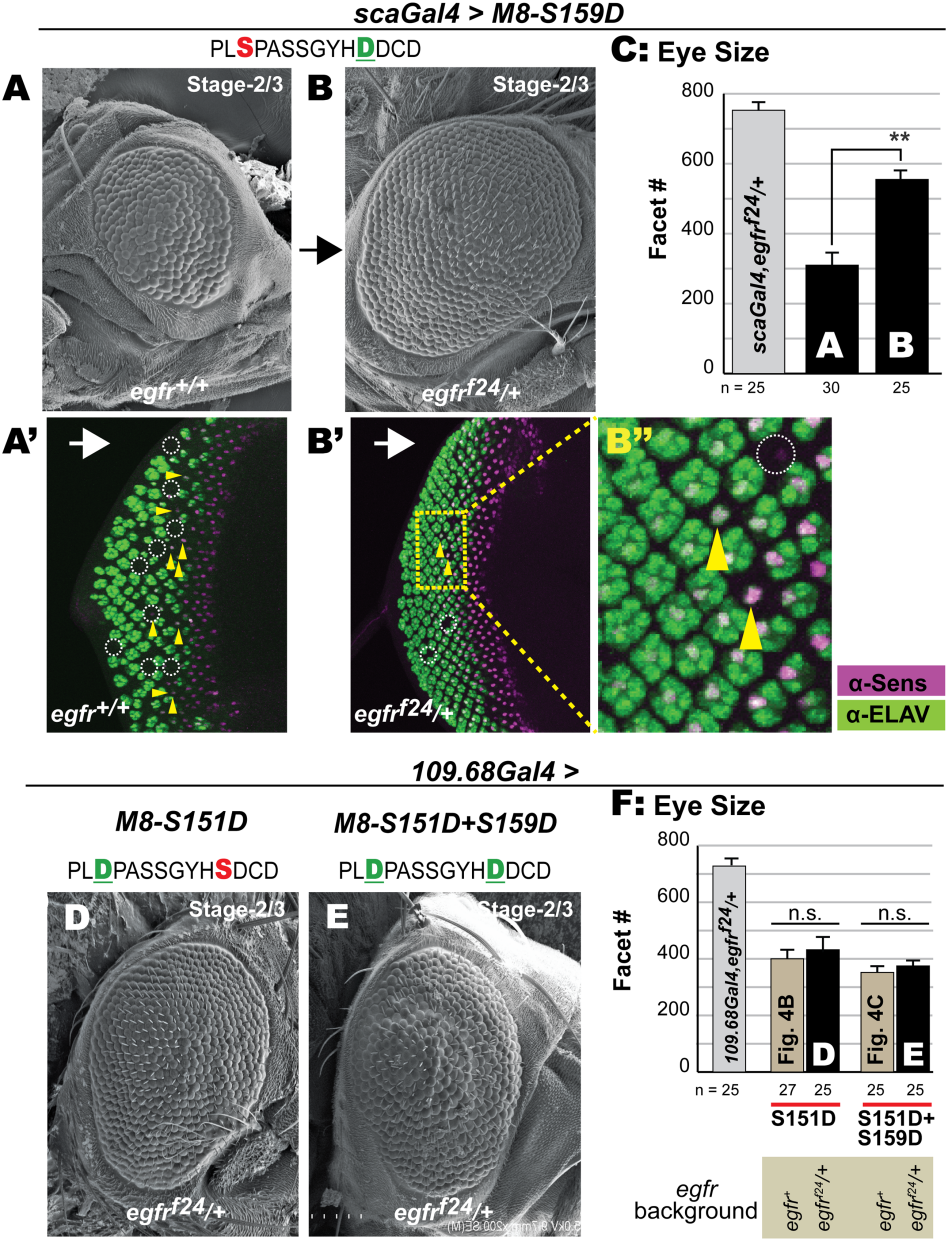
Halved *egfr* dosage mitigates the reduced eye of the CK2 mimic, but not the MAPK mimics. (A-B) Scanning EM of adult eye at 200x magnification. The reduced eye of the CK2 mimic M8-S159D at stage-2/3 (A) is significantly attenuated (rescued) in the *egfr*^*f24*^*/+* background (B). (C) Graph showing eye size; histograms labeled A and B correspond to the eyes in panels A and B. For each genotype, the number of images analyzed for eye size (ommatidial/facet counts) is indicated. Values for genotype shown in panel B were compared to that in panel A (the relevant control) and ** denote P-value < 0.001. Eye size in s*caGal4/+; egfr*^*f24*^*/+* flies was not the control and included to illustrate a WT-eye in this genetic background. (A’-B’) Eye discs of genotypes in A and B were stained to label differentiated R8s (α-Sens) and secondary photoreceptors (α-ELAV). Arrows indicate direction of MF, dotted circles denote missing R8s and secondary photoreceptors, and arrowheads denote recruitment defective R8s. Panel (B”) shows a magnified image of (dashed) yellow box in B’ to highlight rescue of Sens+Elav clusters, although a few R8s fail to recruit Elav+ cells. In contrast, the reduced eye of the MAPK mimic M8-S151D (D) and the dual kinase (CK2+MAPK) mimic M8-S151D+S159D (E) are not rescued in the *egfr*^*f24*^*/+* background. (F) For each genotype, the number of images analyzed for eye size (ommatidial/facet counts) is indicated. Genotypes shown in panels D and E were compared to their corresponding controls, i.e., values from Fig 4B and 4C, respectively, (‘n.s.’ denotes not significant). Eye size in 109-68*Gal4/+; egfr*^*f24*^*/+* flies was not the control and included to illustrate a WT-eye in this genetic background.

Both predictions bear out. The CK2 mimic M8-S159D was expressed with *scaGal4*, given the significant effect on the eye with this stage-2/3 driver. Compared to its effects in *egfr*^*+*^ flies (Fig 7A), the reduced eye of M8-S159D is significantly attenuated in *egfr*^*f24*^*/+* flies qualitatively (Fig 7B) and quantitatively (Fig 7C). Staining of eye discs was next used to reveal the cell specificity underlying restored eye size in *egfr*^*f24*^*/+* flies. Expression of the CK2 mimic M8-S159D in *egfr*^*+*^ flies (Fig 7A’) elicits strong loss of patterned R8s (Sens+ cells), impairs R8 survival (maintenance of Sens levels) and diminished recruitment of secondary photoreceptors (ELAV+ cells). These R8 defects are strongly suppressed in an *egfr*^*f24*^*/+* background (Fig 7B’), although areas with perturbed specification as well as recruitment remain (Fig 7B”). Similar results were obtained in *egfr*^*f2*^*/+* flies (data not shown), diminishing the possibility that a mutation other than that in *egfr* is responsible for the observed modulation.

A similar analysis was conducted with the MAPK-mimic (M8-S151D) and the dual kinase mimic (M8-S151D+S159D), but utilized the driver *109-68Gal4* due to lethality upon expression with *scaGal4* (see Fig 3D). However, the severity of the reduced eye of either variant was indistinguishable in *egfr*^*+*^ versus *egfr*^*f24*^*/+* backgrounds, both qualitatively (compare Figs 7D and 7E to 4B and 4C) and quantitatively (Fig 7F, graph includes values from Fig 4E). Similarly, no modulation was evidenced in *egfr*^*f2*^*/+* backgrounds (data not shown). Staining of eye discs revealed that these two variants elicit R8 defects indistinguishable from those described in *egfr*^*+*^ flies (Fig 5, and data not shown). Thus, eye/R8 defects of M8 variants with a phosphomimetic Asp at the MAPK site are insensitive to halved EGFR/MAPK levels.

We considered, but did not conduct studies on M8 variants in the presence of *Ellipse* (*Elp*^*1*^), an *egfr* gain of function allele, because *Elp/+* flies on their own exhibit a reduced eye [55, 77, 78], thereby limiting our ability to distinguish additive from synergistic effects. As noted above (see Introduction), the CK2 refractory variant M8-S159A was not tested in halved EGFR/MAPK backgrounds, because this variant does not act as a loss of function; rather it elicits a rough eye due to antimorphic effects [46, 47].

### Deletion of the CK2 or MAPK site mimics Asp-variants of M8 at these two kinase sites

**Fig 8:**
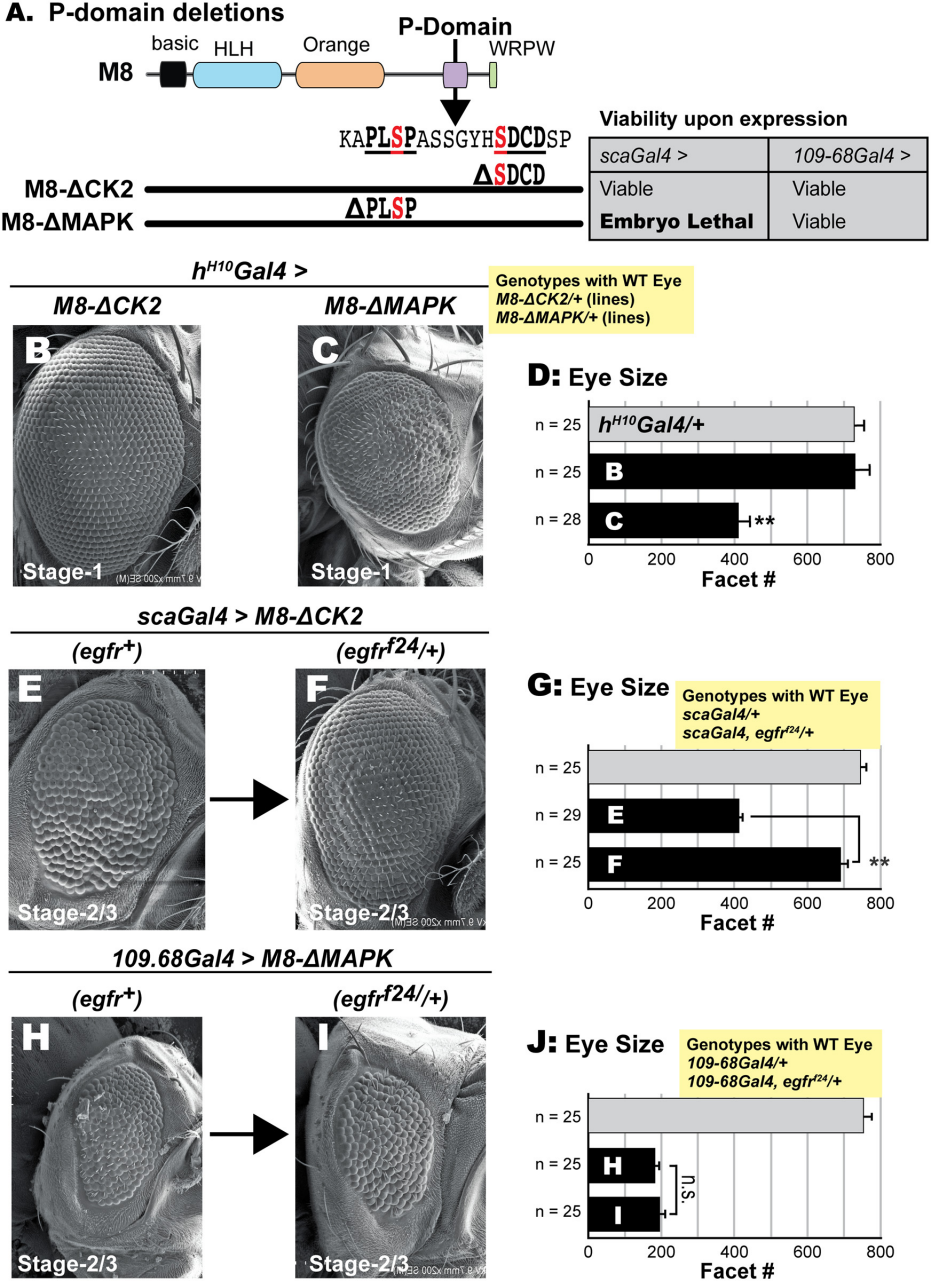
Deletion of the CK2 or MAPK sites in M8 elicits a reduced eye. (A) Deletion variants of the P-domain of M8. M8-ΔCK2 lacks the SDCD motif and M8-ΔMAPK lacks the PLSP motif, but are otherwise full-length. Inset in panel A (shaded grey) shows effects of the two deletions upon expression with *scaGal4* or *109-68Gal4*; note embryonic lethality of M8-ΔMAPK upon expression with *scaGal4*, as also seen with M8-S151D, the Asp variant of the MAPK-site (Fig 3D). Inset (shaded yellow) shows that in the absence of expression M8-ΔCK2 and M8-ΔMAPK transgenic lines have WT eyes (>750 facets with normal hexagonal architecture). Stage-1 expression of M8-ΔCK2 fails to perturb the eye (B) whereas M8-ΔMAPK elicits a reduced eye phenotype (C). (D) Quantitative analysis of eyes in panels B and C, and the relevant control (*h*^*H10*^*Gal4/+*). For each genotype, the number of images analyzed for eye size (ommatidial/facet counts) is indicated. Genotypes shown in panels B and C were compared to controls (*hH*^*10*^*Gal4/+*) and ** denotes P-value < 0.001. Stage-2/3 expression of M8-ΔCK2 elicits a reduced eye with greater severity in *egfr*^*+*^ (E) compared to *egfr*^*f24*^*/+* (F) backgrounds. (G) Quantitative analysis of eyes; values from genotype shown in panel E were compared to that in panel F, and ** denotes P-value < 0.001. In contrast, stage-2/3 expression of M8-ΔMAPK elicits a reduced eye with equal severity in *egfr*^*+*^ (H) or *egfr*^*f24*^*/+* (I) backgrounds. Note that lethality of *scaGal4/+; UAS- M8-ΔMAPK/+* flies (grey inset in panel A), necessitated expression with *109-68Gal4*. (J) Quantitative analysis of eyes; values from genotype shown in panel H were compared to that in panel I (n.s. denotes not significant).Note that grey bars in panels G and J show that the Gal4 driver alone or in combination with *egfr*^*f24*^ (shown in yellow inset above graph) has a wild type eye (>750 facets with normal hexagonal architecture) and was not used as a control for statistical comparisons.

As M8* and MAPK variants (M8-S151D or M8-S151D+S159D) elicit R8/eye defects at stage-1 (Figs 1 and 6), we wished to resolve if the MAPK and CK2 sites directly impose ‘cis’-inhibition on M8, or if the similar stage specificity is purely incidental. Specifically, we sought to determine if modification by CK2+MAPK serves primarily as a conformational switch, rather than for binding repressive cofactors (other than Gro) to the phosphorylated CtD. We tested if deletion of the CK2 or MAPK site renders M8 active, thereby mimicking Asp mutations at these kinase sites. M8-ΔCK2 deletes the known CK2 site S159DCD, whereas M8-ΔMAPK deletes the putative MAPK site PLS151P (Fig 8A). In the absence of expression, neither *M8-ΔCK2/+* nor *M8-ΔMAPK/+* flies exhibit a perturbed eye (yellow inset in Fig 8).

We first tested and found that expression of M8-ΔMAPK with *scaGal4* also elicited embryonic lethality, but no such effect was observed with M8-ΔCK2 (grey inset in Fig 8A). These effects recapitulate results on Asp mutations at the CK2 (Fig 1C) or MAPK sites (Fig 3D). This similarity led us to make three predictions. First, M8-ΔCK2 should more closely resemble the CK2-mimic M8-S159D, i.e., elicit a reduced eye at stage-2/3, but not at stage-1. Second, M8-ΔMAPK should mimic M8-S151D and elicit a reduced eye at stage-1 and stage-2/3. Third, the reduced eye of M8-ΔCK2 should be mitigated by halved EGFR levels, whereas that of M8-ΔMAPK should be insensitive. Indeed, all three predictions bear out. At stage-1 (*h*^*H10*^*Gal4*), M8-ΔCK2 is largely inactive, whereas M8-ΔMAPK elicits a ~50% reduction of the eye field (Fig 8B, 8C and 8D). In contrast, M8-ΔCK2 elicits strong loss of the eye at stage-2/3 (Fig 8E), whose severity is significantly attenuated in an *egfr*^*f24*^*/+* background (Fig 8F), a result also supported by quantitative analysis of eye size (Fig 8G). A similar analysis was conducted on M8-ΔMAPK, but utilized the weaker (stage-2/3) driver *109-68Gal4* to overcome lethality with *scaGal4*. In this case, M8-ΔMAPK elicited a reduced eye of equal severity in *egfr*^*+*^ versus *egfr*^*f24*^*/+* backgrounds (Fig 8H, 8I and 8J).

Thus deletion of the CK2 and MAPK sites elicits eye defects that mimic Asp mutations at these kinase sites. The similar stage-specificity and response to halved EGFR levels lead us to conclude that the two kinase sites directly participate in ‘cis’-inhibition of M8. This control is bypassed by deleting kinase consensus sites (Fig 8), by introducing phospho-mimetic Asp residues (Figs 4–6), or by the removal of the entire CtD, as with M8* (Fig 1B).

### PP2A may target the MAPK site during M8 regulation

**Fig 9:**
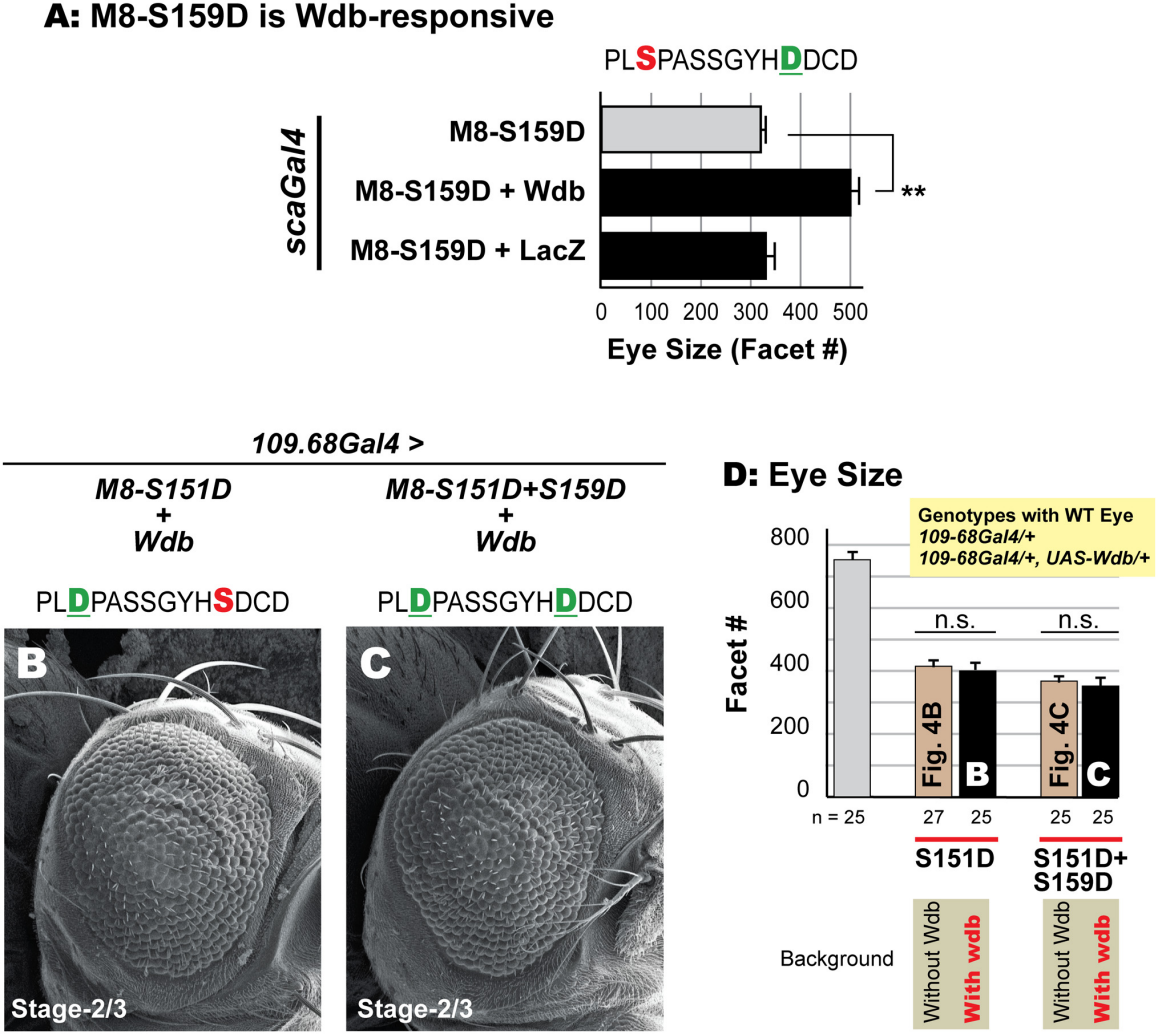
MAPK site in M8 may be a target for the phosphatase PP2A. (A) Co-expression of *widerborst* (*wdb*) rescues the reduced eye of the CK2 mimic M8-S159D, whereas that of *LacZ* elicits no rescue, as previously described [50]. Ommatidial (facet) counts were determined in 20 flies of the indicated genotypes. Values for genotypes upon co-expression of Wdb or LacZ were compared to the corresponding control (*scaGal4/+; UAS-M8-S159D/+*) flies (** denotes P-value < 0.001). In contrast, co-expression of Wdb does not rescue the reduced eye of the MAPK mimic M8-S151D (B) or the dual kinase (CK2+MAPK) mimic M8-S151D+S159D (C). (D) Quantitative analysis of eyes in panels B and C. For each genotype, the number of images analyzed for eye size (ommatidial/facet counts) is indicated. Genotypes shown in panels B and C were compared to their corresponding controls, i.e., values from Fig 4B and 4C, respectively (‘n.s.’ denotes not significant). Note that grey bar in panel D shows that the Gal4 driver alone or in combination with UAS-Wdb (shown in yellow inset above graph) has a wild type eye (>750 facets with normal hexagonal architecture) and was not used as a control for statistical comparisons.

Recent studies from our lab reveal a role for the phosphatase PP2A in birth of patterned R8s [50]. Relevant to the studies described here, overexpression of *widerborst (wdb)*, a PP2A regulatory subunit, suppresses (rescues) the R8/eye defects of the CK2 mimic M8-S159D (Fig 9A). No suppression was seen upon co-expression of *UAS-LacZ*, excluding the possibility that rescue reflects competition between two *UAS*-constructs for a limiting amount of Gal4 protein. On its own, ectopic Wdb does not perturb eye size/patterning (data not shown, and ref [50]). Modulation of the reduced eye of M8-S159D by *wdb* raised the prospect that PP2A targets the MAPK site, rather than that for CK2.

We thus tested if PP2A opposes the effects of EGFR/MAPK signaling, by asking whether co-expression of *UAS-Wdb* modulates the reduced eye of the MAPK-mimic (M8-S151D) or the dual-kinase mimic (M8-S151D+S159D). The reduced eye of either variant upon co-expression of *UAS-Wdb* (Fig 9B and 9C) appeared similar to that in its absence (Fig 4B and 4C). The eye size of either variant in the presence of ectopic Wdb was statistically similar to that in its absence (note that Fig 9D includes values from Fig 4E). Hence, an Asp residue at the MAPK site renders M8 insensitive to Wdb, suggesting that this phosphatase might oppose M8 activation by EGFR/MAPK signaling. In summary, we conclude that the highly conserved MAPK site is important for activation of M8 in a spatially precise manner in the MF, and that this activation is antagonized by PP2A.

## Discussion

The studies described here reveal that the highly conserved MAPK site in E(spl)-M8 is important for repressor activity in the developing eye, a regulation that appears to be dispensable during bristle development. While this overall conclusion is supported by our genetic studies, we note that direct biochemical evidence for modification of M8 by MAPK remains to be established, and neither is it known which of the five Drosophila genes encodes *the* enzyme(s) responsible for modification of the PXS151P motif. Nevertheless, direct demonstration that MAPK targets the similar (PXSP) motif in murine HES6 (Fig 2), a protein also regulated by CK2, raises the likelihood that the predicted MAPK site in E(spl)-M8 is of functional significance. Although our studies are based on the Gal4-UAS approach, we do not think that they reflect developmental abnormalities simply due to mis-expression, because a) endogenous *m8* is expressed in the MF and *E(spl)D* potently impairs R8 birth and eye development [43], and b) these Gal4-drivers have been widely used to evaluate activities of E(spl) proteins in the developing eye and elsewhere [28, 29, 43, 65, 79, 80].

Specifically, we have used the eye and bristles as in vivo ‘readouts’ to evaluate the activities of M8-variants with Ala/Asp at the MAPK (and CK2) sites in wild type and *egfr* mutant backgrounds (Figs 1 and 3–7). Along with studies on deletion variants of M8 (Fig 8), these findings are consistent with a role for EGFR/MAPK signaling in controlling M8 repression of Ato, and implicate both kinase sites (CK2 and MAPK) in ‘cis’-inhibition. Finally, our analysis of PP2A-Wdb (Fig 9) raises the prospect that this phosphatase, previously implicated in lateral inhibition [49, 50, 81, 82], targets the MAPK site of M8.

### Regulation of E(spl) members by multi-site phosphorylation

The strong conservation of sites for these two kinases in M7, M5 and Mγ reinforce our view that this regulation has a broader impact on E(spl) proteins than has been recognized. The E(spl) members have been viewed as partially redundant dosage-dependent effectors of Notch signaling. This view, reflects the observations that the manifestation of neurogenic phenotypes needs simultaneous loss of multiple members [33, 34, 83], and that neural (bristle) development is blocked by ectopic expression of any of the seven E(spl) proteins [28, 29, 43, 79, 84–86]. However, this ‘dosage-only’ model for repression has not borne out in the developing eye. For example, even though M8, Mγ or Mδ are normally expressed in the MF, ectopic expression of only Mδ (at a double dose) impairs R8 birth [43, 46, 80]. Consequently, it has been suggested that these proteins may be ‘qualitatively’ different [80]. In light of our studies on regulation of M8 by CK2 and MAPK, it seems likely that distinct tissue-specific regulatory mechanisms control repression by E(spl) proteins.

### Multi-site phosphorylation and spatial control over M8 activation in the eye

**Fig 10:**
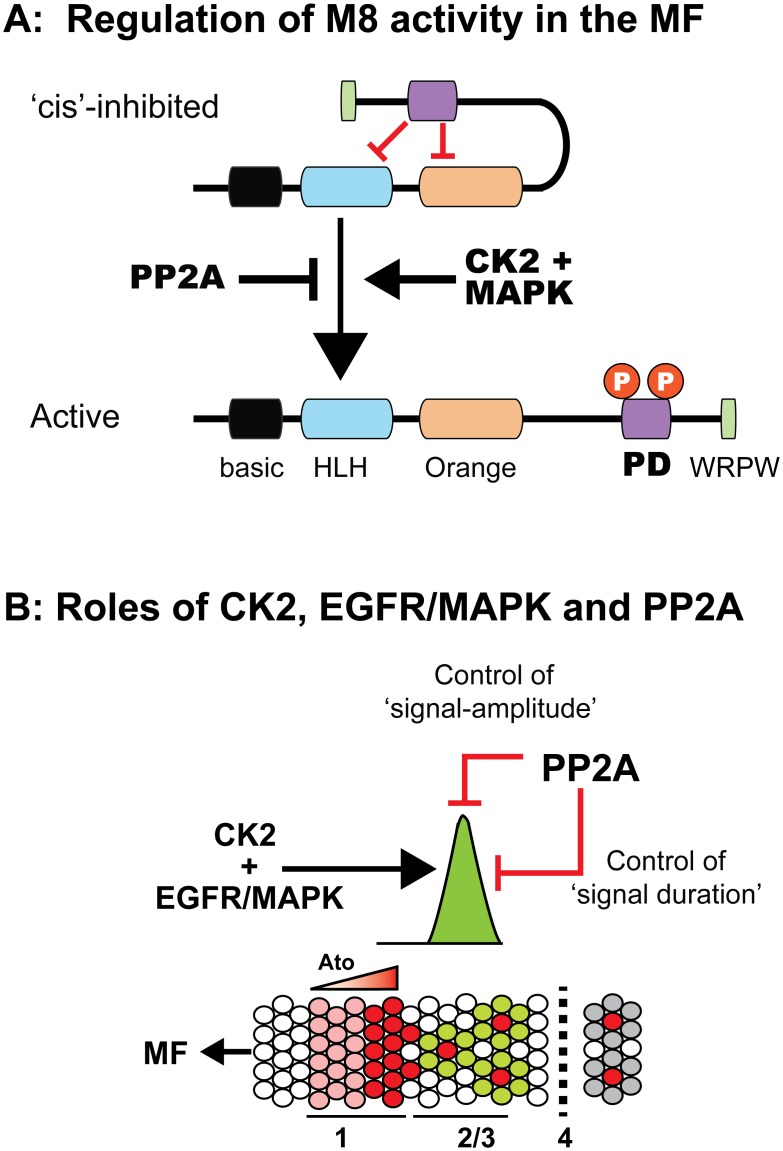
Regulation of M8 activity by reversible phosphorylation. (A) In the ‘cis’-inhibited state, the CtD interacts with the HLH and/or Orange domains thereby blocking M8 repression of Ato. Phosphorylation by CK2 and MAPK serves as a conformational switch that converts M8 into an Ato repressor, whereas PP2A mediates inactivation. (B) Model for spatial and/or temporal regulation of M8 activity at stage-2/3 of the MF. EGFR/MAPK signaling ensures that M8 activation does not occur until stage-2/3 of the MF, thereby allowing *ato*-auto-activation to raise Ato levels to a threshold sufficient for the R8 fate. In this case, the phosphatase PP2A targets M8 to control either the ‘amplitude’ or ‘duration’ of active M8.

The studies we describe here expand on our understanding of the role of two kinase sites within the P-domain of M8. Our data suggest a revised model wherein conversion of ‘cis’-inhibited M8 into an active repressor of Ato depends upon phosphorylation of the CK2 and MAPK sites, and that the precise activation of this Ato repressor at stage-2/3 of the MF occurs in response to EGFR signaling (Fig 10A and 10B). If so, it would suggest that EGFR signaling, which is necessary and sufficient to activate MAPK at stage-2/3, is required for inhibitory effects of Notch, and without which R8 patterning would be perturbed, as has been well documented (see Introduction). The regulation of M8 activity by PP2A-Wdb raises the prospect that this phosphatase may play two roles, which we do not consider to be mutually exclusive (Fig 10B). By antagonizing modification at the MAPK site, PP2A may control the amount of active M8, or drive inactivation immediately upon the completion of lateral inhibition, i.e., the emergence of single R8s. The former regulation would control *‘signal amplitude’* (amount of active M8), whereas the latter would control *‘signal duration'* (how long M8 remains active). Our studies, which do not discriminate between these possibilities, nevertheless reveal that multi-site phosphorylation controls repression by M8, extending the proposal that ‘timing’ is of essence to Notch signaling [87, 88].

We note that our analyses do not reveal the order of M8 phosphorylation, which can only be revealed by antibodies that recognize not only specific isoforms but also specific (CK2, MAPK or dual) phosphorylated states. Our efforts to raise such antibodies have not been successful (Bose and Bidwai, unpublished), as they display cross-reactivity with unmodified M8 and other members with conserved CtDs (M5, M7, Mγ). We discuss the implications of our findings in the context of two pathways, Notch and EGFR, which are vital not only for R8 ontogeny, but also for later stages of retinal histogenesis.

### Notch and the specification of the R8 cells

The key role of Notch as *the* driver for lateral inhibition and cell fate determination is indisputable (reviewed in [89–95]). Detailed studies have shown that loss of *Su(H)* or the *E(spl)C* impairs lateral inhibition and elicits the specification of ‘twinned’ R8s and bristle SOPs. The developmental outcome of loss of Notch is more complicated, in particular, at the onset of retinal histogenesis, given dual roles of this receptor for R8 birth. As stated above (see Introduction), Notch is first needed for ‘proneural induction’ and immediately after for lateral inhibition. The molecular mechanism(s) underlying these antagonistic functions has, to our knowledge, remained undefined. Spatially controlled phosphorylation of M8 may function as a *‘time-delay-circuit’*. Without this delay, active M8 would impair *ato* up-regulation, and Ato levels in pre-R8 clusters would fail to achieve the threshold necessary to adopt the R8 fate. Indeed, RNA in-situ hybridization reveals that *m8* is expressed early in the MF and *E(spl)D* largely enhances transcript levels through stabilization [96], suggesting that the R8/eye defects of *E(spl)D* are unlikely to reflect earlier than normal expression. Constitutively active M8* would disrupt the Ato-Da cooperativity [97]. The loss of Ato up-regulation seen with *E(spl)D* or stage-1 expression of M8* seems consistent with our model, and underscores the prescient suggestion of Giebel and Campos-Ortega [29] that this region (the CtD) may impose control over repression.

This, in turn, raises the question on why MAPK is dispensable for M8 regulation in the bristle? While the answer(s) to this question remain unclear, we speculate that this may reflect intrinsic differences in Notch functions in retinal versus bristle development. In the latter sensory organ, expression of the proneural activators encoded by the *achaete scute Complex* (*ASC*) occurs in response to pre-pattern factors [1], i.e., is Notch-independent. This major difference from biphasic Notch signaling during genesis of the R8 cells, might explain why retinal patterning would require spatial control of M8 activity (through MAPK, see above). In addition, the R8s immediately initiate recruitment of secondary photoreceptor pairs (R2/R5, R3/R4, R1/R6 and the R7), all of which require repeated rounds of Notch signaling and E(spl) activities, and occurs at the rate of 1 column of ommatidia every 2 hours. This is in contrast to the bristle SOP, which is selected from a PNC in the third larval instar, but the post-SOP asymmetric divisions that generate the neuron, sheath, socket and shaft cells occupy ~24 hours of pupal development. The shorter time-frame in a dynamic MF, combined with closely timed and repeated Notch signaling may thus require tight spatial/temporal control, ideally suited for regulation through phosphorylation. An alternative (and equally likely) possibility is that repression by E(spl)-M8 involves multiple and distinct tissue specific mechanisms such as repression through DNA-binding, direct binding (and sequestration) of proneural proteins, and/or the recruitment of the co-repressor Gro. Evidence for such multiple mechanisms has been reported in S2-cell based assays and the developing wing [98], the MCs [29], and the eye [43]. Moreover, this alternative is supported by our finding that MAPK refractory variant M8-S151A+S159D elicits MC loss with potency similar to that of the CK2-mimic M8-S159D (Fig 4).

### EGFR and the specification of the R8 cells

Our work appears consistent with analysis of the *Elp* allele of *egfr* by Baker and coworkers who have proposed that ‘some level’ of EGFR pathway activity is necessary for lateral inhibition to occur in a timely manner [55],. While loss of *egfr*, *ras* or *raf* indeed elicit supernumerary R8s, no such effects are seen upon loss of *pnt* or *yan*. Analysis of R8 birth in clones of MAPKs has been precluded by cell lethality [56]. Nevertheless, active MAPK has been directly demonstrated (biochemically) at stage-2/3 of the MF. However, unlike canonical EGFR/MAPK signaling, dp-ERK in the eye disc remains cytosolic [52] precluding phosphorylation of Pnt or Yan, which are constitutively nuclear [58], and whose loss does not perturb R8 patterning. The identity of the protein(s) targeted by this active cytosolic MAPK has remained unknown. Do our studies suggest that active MAPK regulates M8 in the cytosol only after which active M8 enters the nucleus to repress Ato? While the answers to this question will necessitate phospho-M8 specific antibodies, we note that the default location of HES6, the human homologue of M8, is also cytosolic [99], and nuclear localization of this repressor is closely coupled to induction of (myogenic) differentiation, suggesting that the nucleus is not the default location of (at least) this HES repressor.

How might MAPK precisely regulate M8 activity? Our studies on deletion variants of M8 and their MF stage-specificity provide clues. We have previously proposed that inactive M8 is ‘cis’-inhibited [46, 100]. That the deletion of the CK2 site closely mimics the effects of an Asp substitution (M8-S159D, see Fig 8), suggests that the CK2 site itself participates in protein-protein contact(s). However, this variant fails to elicit a reduced eye (or perturb R8 birth) at stage-1 of the MF, which can only be engendered by deletion of the MAPK site (Fig 8). The virtually identical outcomes of Asp mutations at the CK2 and/or MAPK sites or by deletion of their consensus sites, diminish the likelihood that phosphorylation controls binding of cofactors essential for repression. More likely, phosphorylation by CK2+MAPK is structurally coupled to overcoming ‘cis’-inhibition (Fig 10A). This regulation may have a greater role during R8 birth, when Ato repression by E(spl)-M8 is thought to depend largely on direct protein-protein interactions with minimal contributions by DNA-binding or Gro-recruitment [43, 46, 101]. One question that has remained unanswered is what triggers the modification of M8 by CK2? This kinase does not respond to extracellular or intracellular signaling pathways (reviewed in [102–105]), yet regulates precise temporal processes such as cell cycle progression and the circadian clock [106–110]. Moreover, CK2 does not modify M8 on its own because co-expression of CK2 and M8 does not pheno-copy the effects of the CK2-mimic M8-S159D [49]. In any event, given that MAPK activation (in the MF) is exclusively dependent on EGFR pathway activation, ‘spatial’ control could well be mediated by EGFR signaling. This spatial control of M8 activity is mechanistically similar to recent studies that PKA mediated phosphorylation inactivates DNA-binding by Ato (and other proneural proteins such as Scute and Neurogenin2) thereby controlling the duration of their transcriptional activities, and that perturbing the site for this kinase elicits neuronal cell fate defects [111, 112].

These studies for the first time demonstrate a developmental context in which the biological role of the MAPK site in E(spl)-M8 has been revealed. As shown in Fig 10, coordinated functions of several kinases (CK2 and MAPK) and the phosphatase (PP2A) control when and where M8 repressor activity manifests. These modifications may balance the amplitude of the M8 signal or the duration of its repressive functions. Such control over M8, raises questions on other developmental programs such as myogenesis, oogenesis, etc., known to require repression through Notch and E(spl) proteins. Future studies will be needed to resolve which MAPK gene(s) participate in the post-translational regulation of E(spl)-M8 activity in the developing eye, and how and where else this regulatory mechanism operates to fine tune the inhibitory effects of Notch signaling.
